# Mesenchymal Stem Cells in Gastric Cancer: Vicious but Hopeful

**DOI:** 10.3389/fonc.2021.617677

**Published:** 2021-05-11

**Authors:** Yuyi Li, Xingwei Zhong, Yunzhu Zhang, Xinliang Lu

**Affiliations:** Department of Gastroenterology, The Second Affiliated Hospital, Zhejiang University School of Medicine, Hangzhou, China

**Keywords:** gastric cancer, mesenchymal stem cells, tumor tropism, reprogramming, tumor stroma, drug delivery, targeted therapy

## Abstract

Tumor progression depends on the collaborative interactions between tumor cells and the surrounding stroma. First-line therapies direct against cancer cells may not reach a satisfactory outcome, such as gastric cancer (GC), with high risk of recurrence and metastasis. Therefore, novel treatments and drugs target the effects of stroma components are to be promising alternatives. Mesenchymal stem cells (MSC) represent the decisive components of tumor stroma that are found to strongly affect GC development and progression. MSC from bone marrow or adjacent normal tissues express homing profiles in timely response to GC-related inflammation signals and anchor into tumor bulks. Then the newly recruited “naïve” MSC would achieve phenotype and functional alternations and adopt the greater tumor-supporting potential under the reprogramming of GC cells. Conversely, both new-comers and tumor-resident MSC are able to modulate the tumor biology *via* aberrant activation of oncogenic signals, metabolic reprogramming and epithelial-to-mesenchymal transition. And they also engage in remodeling the stroma better suited for tumor progression through immunosuppression, pro-angiogenesis, as well as extracellular matrix reshaping. On the account of tumor tropism, MSC could be engineered to assist earlier diagnosis of GC and deliver tumor-killing agents precisely to the tumor microenvironment. Meanwhile, intercepting and abrogating vicious signals derived from MSC are of certain significance for the combat of GC. In this review, we mainly summarize current advances concerning the reciprocal metabolic interactions between MSC and GC and their underlying therapeutic implications in the future.

## Introduction

Gastric cancer (GC) is one of the most refractory malignancies with high morbidity and mortality. Updated statistics indicates that GC is the fifth frequently diagnosed cancer with over 1,000,000 new cases and the third leading cause of cancer-associated morbidity, an estimated 783,000 deaths worldwide (1). Helicobacter pylori (Hp) eradication, gastroscopy, and endoscopic treatment have reduced the risk of developing GC, as well as provided better long-term health-related quality of life for patients with early GC (2, 3). Regrettably, a larger population of people is already in the advanced stage at the first diagnosis for rarely present symptoms. Although a combination of surgical resection with adjuvant chemotherapy is the preferential option for advanced GC, the survival outcome stays disappointing, dropping a median overall survival of 10-12 months (4, 5). Hence, searching for new strategies, such as immunotherapy, targeted therapy, and making clinical transformation remain urgently needed.

Of note, the occurrence and development of tumor cannot be isolated from the tumor microenvironment (TME). The surrounding microenvironment “soil” is to facilitate the survival and thriving of tumor cell “seed” *via* substantial reciprocal crosstalk between cell-cell or cell-non-cell components (6). TME is generally a complex network and largely composed of the stroma of tumor, covering mesenchymal stem cells (MSC), cancer-associated fibroblasts (CAF), endothelial cells (EC), pericytes, immune cells, vasculature together with the extracellular matrix (ECM) surrounding the cancerous tissue (7–9). Contrast to stroma under normal physiological conditions, which encompasses structural and supportive framework to maintain the stability of tissues and suppresses cancer proliferation, the tumor stroma is in an active state and has been documented to be firmly correlated with the aggressiveness and unfavorable clinical outcomes of a spread of malignancies including GC (10–12). A cohort study with 583 gastric adenocarcinomas demonstrated that stroma-rich patients tend to acquire a worse 5-year prognosis than stroma-poor ones, no matter in intestinal or diffuse histological phenotype (13). Other studies suggest tumor-stroma ratio a reliable prognostic indicator to optimize risk stratification in GC for the ability to quantify the effect of tumor-stroma interactions on tumor biology (14, 15). Such evidence sheds light on the important role of stroma for tumor development, which would arise novel anti-cancer strategies focusing not restrictive on cancer cells.

Among the stromal cells, MSC, a population of non-hematopoietic cells with self-renewal capacity, multi-differential potential, and immune-modulatory property, have received recent attention as a key contributor in directing tumor behavior and TME remodeling. MSC are spindle-shaped cells and capable of forming colonies when originally isolated from the hematopoietic microenvironment of bone marrow (BM), also named colony-forming unit-fibroblast (CFU-F) (16). Then the International Society for Cellular Therapy (ISCT) enacted the minimal criteria for better isolation of these cells: firstly, be plastic-adherent; secondly, highly express (>95%) surface molecules of CD73, CD90, CD105 and lack expression of (<2%) CD45, CD34, CD14, CD11b, CD79α, CD19 and HLA-DR; thirdly, hold the ability to differentiate into osteoblasts, adipocytes, and chondroblasts *ex vivo* (17). Over time, other surface antigens, like CD271, STRO-1, CD106 and CD146 come to be accepted and recognized for MSC identification (18, 19). MSC used to arouse excitement for regenerative medicine owing to they can quickly engraft to inflammatory cytokines or chemoattractant gradients produced by injured tissue and organs to exhibit their tissue healing functions, alone or in combination with other methods (20, 21). With the hypothesis “tumor equates wound that never heals” arises, the function of MSC in tumors has been realized to parallel the role of MSC in wound healing that actively promotes MSC from bone marrow or adjacent tissues to mobilize into the TME (22, 23). In a mouse model of hepatocellular carcinoma (HCC), MSC possess distinct homing profiles and contribute to a significant rapid depletion from circulation in cancerous condition (24). The result is consistent with findings in the patients with GC showing that an intensified peripheral trafficking of MSC in comparison to healthy individuals; and the egressed MSC are commonly aggregated in tumor bulks over adjacent normal tissues (25, 26). That throws the great interest to explore the possible roles of MSC in tumor progression and aggressiveness which previously may be neglected in GC-stroma interactions. In the following, we mainly cover recent advances in the interactions between MSC and GC, the role of MSC in rewiring the nearing cancer stroma, and potential underlying mechanisms. Furthermore, fresh boundaries regarding the potential application of MSC in GC are also within our discussion to inspire more preventive and therapeutic strategies.

## The Recruitment and Reprogramming Course of Naïve MSC Into GC-MSC

### The Tropism and Remodeling of Naïve MSC

GC as well as its progression niche is a reservoir of cytokines, chemokines, growth factors that specifically drive the tropism and motility of BM-derived cells including MSC. Under the co-culture system, the migratory ability of BM-MSC could be raised up to two- to threefold because of the high expression of CXC receptor 2 (CXCR2) in response to CXC ligand 1 (CXCL1), a chemokine stimulating factor released from cancer cells (26). Except for this, chemotactic signals derived from GC such as C-C chemokine ligand 19 (CCL19) and CXCL12 would also augment the migration potential of MSC to cancer in a dose-dependent manner, while the concrete mechanisms still to be further investigated (27). On the other hand, it is important to note that Hp infection would accelerate the trafficking of MSC into the stomach at the early stage of carcinogenesis. Hp-mediated chronic inflammation of gastric epithelial cells would significantly increase the secretion of tumor necrosis factor-alpha (TNF-α), an appreciable molecule in stimulating MSC migration in Nuclear Factor-kappa B (NF-κB)-dependent manner (28). Subsequently to being recruited, the naïve MSC [mainly refers to BM-MSC or MSC isolated from adjacent non-cancerous tissue (GCN-MSC)] were relentless to be educated by GC cells to become specialized ones equipped with the tumor-supporting capacity (29). Moreover, the immunomodulatory function of MSC could also be modified *via* the activation of the NF-κB signaling pathway, to strengthen their activation ability to immune cells (30). Intriguingly, a recent finding suggested that BM-MSC incorporated into metastatic lymph node microenvironment could be reprogrammed by cancer cells. Yes-associated protein (YAP) activation elicited by exosomal Wnt5a from lymph node-derived GC cells, was verified pivotal for their reprogramming into cancer-associated MSC (31).

### Characterization of GC-MSC

Evidence incline to depict that GC-MSC and naïve MSC share equivalent spindle-shaped morphology, similar surface antigens and stem cell-related gene expression. GC-MSC are positive for CD13, CD29, CD44, CD73, CD90 and CD105, but negative for CD14, CD31, CD34, CD38, CD45, CD71, CD133 and HLA-DR, among which the expression of CD105 was strongly associated with the poor prognosis of GC patients (12, 32–34). Another study denoted that the higher co-expression of CD29 and CD90 are more commonly seen in GC-MSC than GCN-MSC, and was correlated with more advanced pathological stage, worse disease-free survival and overall survival (35). In comparison with naïve MSC, GC-MSC seem to be on a less quiescent stage, ultrastructurally, phenotypically and functionally. GC-MSC typically feature greater number of cell organelles, such as mitochondria and endocytoplasmic reticulum, and higher expression of proliferation-related genes, all of which are coincide with their greater proliferative potential (32). Besides, GC-MSC display a stronger intensity of reactive stroma cell markers including fibroblast activation protein (FAP) and α-smooth muscle actin (α-SMA) (36). In virtue of the reprogramming by cancer cells, GC-MSC exhibit a higher secretion of inflammatory cytokines than naïve MSC, e.g., interleukin-6 (IL-6), IL-8, transforming growth factor β1 (TGF-β1), ect, which in turn display superior efficiency in facilitating cancer cell growth, invasion, migration and tumorigenesis *in vitro* and *in vivo* (36, 37).

## MSC Impact on GC Cells

### Aberrantly Activate Oncogenic Cell Signaling

**Figure 1:**
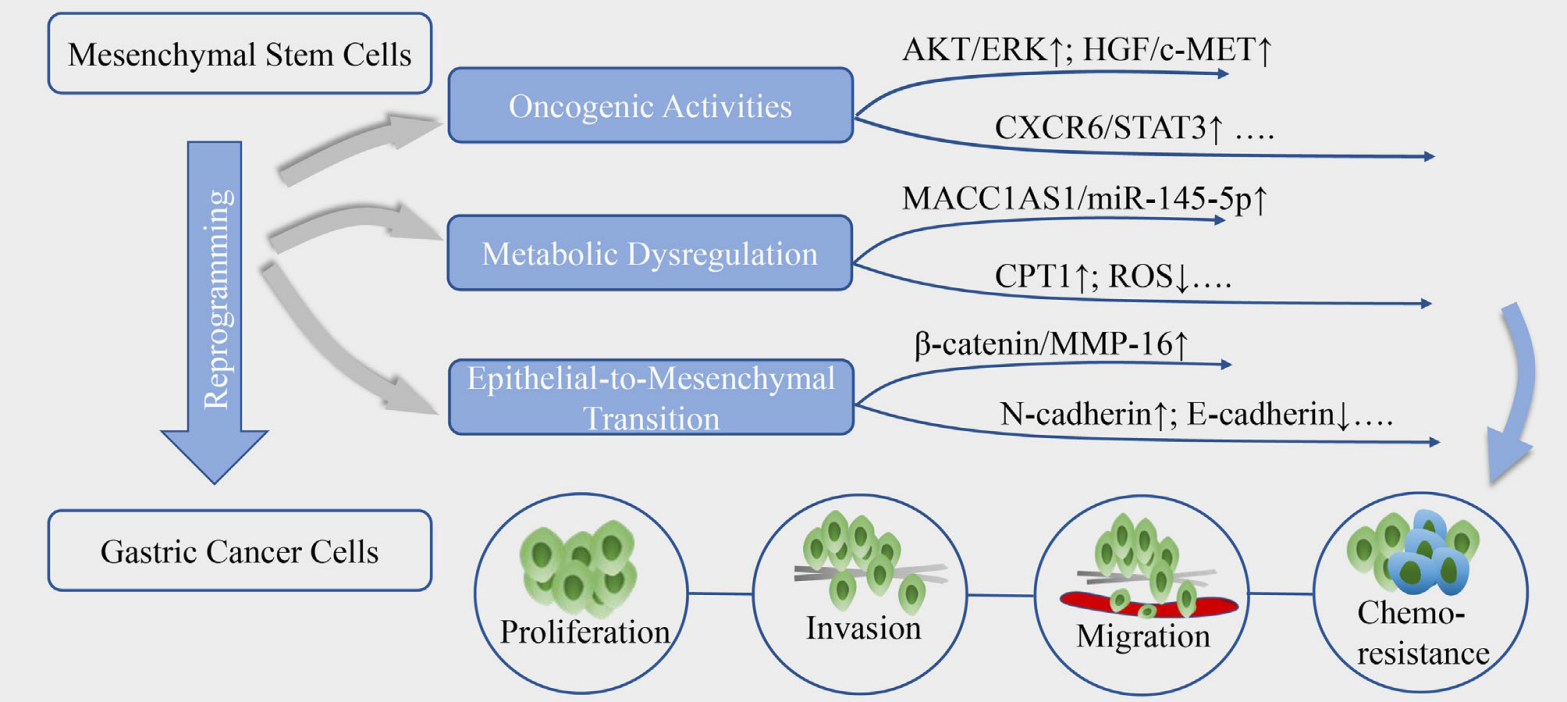
The reprogramming of GC by MSC. MSC could reprogram the biological activities of gastric cancer cells, mainly through aberrantly activate oncogenic signals, metabolic reprogramming and epithelial-to-mesenchymal transition, contributing to cancer cells proliferation, invasion, migration and resistance to chemotherapy.

MSC are capable of reprogramming GC cells to orchestrate the proliferation, invasion, migration and chemoresistance, *via* stimulation of oncogenic signaling pathways associated with aberrantly growth or transforming (**Figure 1**). The enhanced secretion of IL-8 from GC-MSC has been linked to cancer progression by inducing the activation of protein kinase B (AKT) and extracellular signal-regulated protein kinase (ERK) 1/2 signaling pathway (37). Moreover, GC-MSC could robustly express hepatocyte growth factor (HGF) as ligand of c‐MET to trigger phosphorylation of its downstream signaling cascade in cancer cells, and aberrant HGF/c-MET axis has been well-established to be critical for GC progression (38). Recently, researches highlighted the crucial mediator of CXCR6 and signal transducer and activator of transcription 3 (STAT3) pathway, which suggest a prominent production of CXCL16 in MSC via the activation of Wnt5a/Ror2 signaling axis, in turn, activates its corresponding receptor CXCR6 to increase the expression of Ror1 via the activation of STAT3, eventually resulting in the promotion of proliferation and migration of GC cells (39, 40). In addition to these, emerging studies have uncovered the oncogenic potential of platelet-derived growth factor (PDGF)-DD/PDGFR-β axis (41) and YAP/β-catenin signaling (42) in the MSC-induced cancer initiation and progression, and blocking or interference of these signals has designated a certain positive significance for the treatment of GC.

Exosomes prove a kind of small lipid bilayer membrane vesicles delivering intercellular communication bioactive molecules, like miRNAs, long non-coding RNAs (lncRNAs), and proteins to reshape the biological behavior of adjacent cells or remote targets of the body (43, 44). As research conducted by Ji et al. (45), the exosomes from MSC can trigger the calcium-dependent protein kinases (CaM-Ks) and its downstream RAF/MEK/ERK signaling cascade, elevating the expression of multi-drug resistant proteins in cancer cells to counteract 5-fluorouracil (5-FU) induced cell apoptosis, however, the exact molecules in exosomes that mediate this effect have yet been identified. Ubiquitin-protein ligase E3 component n-recognin 2 (UBR2) is a kind of protein that accounts for ubiquitination and degradation, was found to highly aggregate in exosomes of especially p53 deficient BM-MSC, could be internalized into GC cells and induce the activation of Wnt/β-catenin pathway to promote cell proliferation, migration and stemness maintenance (46). In addition, evidence has clarified that miRNAs are deregulated in exosomes of GC-MSC, to be transferred to transcriptionally modulate cancer aggressiveness, of which miR-221 deregulation has been linked with various tumorigenic pathways (47, 48).

### Dysregulate Metabolic Plasticity

Metabolic reprogramming has been proposed as a new hallmark for cancer progression. Emerging discoveries highlight that MSC could dysregulate cell metabolism to confer GC cells stemness and tolerance to drug stress (**Figure 1**). He et al. (35) proposed that TGF-β1 secretion by MSC co-opt TGF-β receptors in GC cells could raise the expression level of lncRNA MACC1-AS1, which have sponge interaction with miR-145-5p to boost the expression of CPT1, the fatty acid oxidation (FAO) speed-limiting enzyme, subsequently decreasing reactive oxygen species (ROS) production and cell apoptosis under 5-FU and oxaliplatin. Mechanistically, MACC1-AS1 also is found to augment the expression of MACC1, an oncogene and a poor prognosis marker in GC, to elevate glutathione and nicotinamide adenine dinucleotide phosphate (NADPH) levels and sustain a lower ROS load under metabolic stress (49). In another study, MSC-induced lncRNA histocompatibility leukocyte antigen complex P5 (HCP5) in GC cells was demonstrated to serve as a miR-3619-5p sponge to facilitate FAO *via* the transactivation of CPT1, thereby alleviating the cell cycle arrest effect of cancer cells caused by chemotherapeutics (50). Besides, the antagonist of CPT-1 could remarkably reverse the MSC-induced tumor growth under FOLFOX regiment treatment *in vivo* (35), indicating that deregulated FAO could be a key regulator of MSC-mediated chemoresistance in GC and a potential target for anti-resistance interventions.

### Elicit Epithelial-to-Mesenchymal Transition (EMT)

Phenotypic transition occurs in GC cells when close physical contact with MSC as well (**Figure 1**), they adopt mesenchymal phenotype including longitude ridging, ruffled membranes and finger-like extensions, concomitantly with increased level of α-SMA, N-cadherin, vimentin and Snail and repressed expression of cellular adhesion molecules especially E-cadherin (51–53). EMT is a dynamic process and the mesenchymal traits endow the malignant cells migratory and invasive capacities, and the susceptibility to cancer intravasation and metastasis. The paracrine signals of MSC also induce EMT to promote transendothelial migration, mechanistically, dependent on high expression of snail, twist, β-catenin and matrix metalloproteinase-16 (MMP-16) (52). Moreover, the activation of PI3K/AKT signaling pathway was shown to be linked with the process (51). Intriguingly, researchers found cell fusion maybe one of the underlying mechanisms in the MSC-primed EMT. Hybrids acquire the mesenchymal and stemness proteins, enhanced proliferation and migration potential during a physical fusion event with MSC (54). Notably, hybrids generated by MSC and immortalized non-tumorigenic human gastric epithelial cells also undergo EMT and are vulnerable to be malignant transformation, which makes a difference in cancer initiation (55).

## MSC Remodeling the GC Stroma

### Immunosuppression Potential of MSC

**Figure 2:**
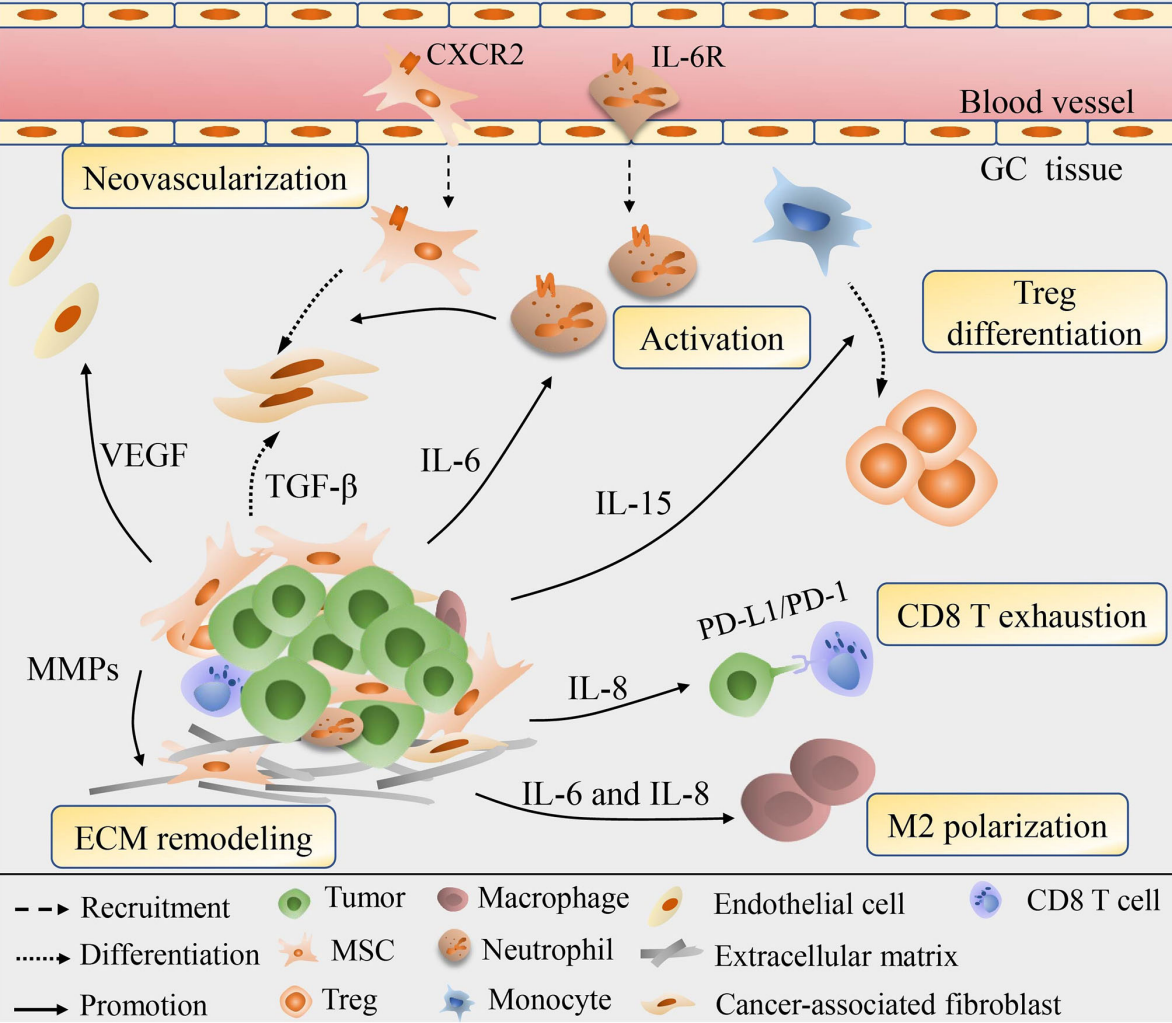
Schematic diagram for the role of MSC in the remodeling of GC stroma. MSC could be recruited into cancer stroma and are involved in the continuous updating and transformation of stroma components. *Via* secretion of various cytokines, e.g., IL-6, IL-8, IL-15, MSC modulate the Treg differentiation, macrophage polarization, neutrophil recruitment and activation, exhaustion of CD8 T cells, thus offering local immune-suppressive milieu. MSC could also secret VEGF and MMPs to induce neovascularization and ECM degrading, or directly differentiate into CAF to exhibit synergistic effect in remodeling cancer cells and stroma.

Not only function directly back upon tumor cells to boost the growth and progression, but MSC are also involved in the continuous updating and transformation of stroma components, for the aim of enhancing their tumor-supporting roles and accommodating the rapid metabolic process of tumors (**Figure 2**). The immunomodulatory property of MSCs has been well exploited to prevent and treat severe graft-verse-host diseases (56). With the reprogramming of GC, MSC incorporation engraftment the TME could modulate the differentiation, polarization or anergy of immune cells thus offering local immune-suppressive milieu. GC-MSC, mainly by extracellular cytokines secretion, such as IL-15 contained in the conditioned medium (CM) could predispose peripheral blood mononuclear cells (PBMC) to skew their differentiation into regulatory T (Treg) population (57). The balance of Treg and Th17 subsets defines a key regulator for immune homeostasis for they exert the opposite immune-modulatory functions (58). Consistently, a study conducted by Wang et al. (59) revealed that by the joint activation of GC-MSC, the enhanced differentiation of Treg subsets and the suppressed Th17 cells proliferation can reverse the tumor-inhibitory effect of PBMC to significantly improve GC growth potential and facilitate liver metastases formation *in vivo*. Li and colleagues (60) have shown that GC-MSC also perform an intricate crosstalk with macrophages to drive the conversion of macrophages toward alternatively activated, immunosuppressive M2 phenotype, which exhibit a higher potential in promoting GC invasion and metastasis than that of GC-MSC. Importantly, the M2 phenotype has been manifested to play extensive roles in immune tolerance, neo-angiogenesis, pre-metastatic niche formation for GC advancement (61). Mirroring macrophages, the IL-6 enriched in the GC-MSC-CM can stimulate the phosphorylation of STAT3 and ERK1/2 in neutrophils, promoting their recruitment and activation into a pro-tumor phenotype that would finely cooperate with GC-MSC to synergistically prompt cancer migration and angiogenesis (34). Intriguingly, GC-MSC could induce the anergy and silence of immune potency through the secretion of IL-8 to upregulate the expression level of PD-LI in GC cells that drive the exhaustion of CD8 T cells, resulting in immune resistance and contributing to GC progression (62). Emerging research spots that the immunophenotype of GC-MSC are also influenced by the CD4 T cells *via* the p-STAT3 signaling pathway to boost cancer growth rate-promoting role of GC-MSCs, highlighting that TME is a huge complicated signaling network in which tumor cells, immune cells, MSC and other components are in a multi-angle communication to facilitate cancer progression (63).

### Vascularization and ECM Remodeling

Neovascularization provides favorable conditions for tumor invasion and metastasis and is therefore considered a marker of poor prognosis. Under the pretreatment of GC-CM *in vitro*, MSC would elevate the expression of vascular endothelial growth factor (VEGF), macrophage inflammatory protein-2 (MIP-2), TGF-β1, IL-6, and IL-8, among them VEGF being highly angiogenic works prominently in vascular development (37). Consistent with the prior finding, Feng et al. (64) proposed that VEGF production, in collaboration with NF-κB signaling in GC-MSC, could induce angiogenesis through driving the human umbilical vein endothelial cells (HUVEC) tube formation, suggesting the active involvement of GC-MSC in tumor neovascularization, while the inherent regulation mechanisms in VEGF/NF-κB signaling remains unquestioned. MSC are observed to trans-differentiation into EC with the induction of basic fibroblast growth factor (bFGF) or VEGF, and RNA chip result indicates the intrinsic epigenetic modifications of MSC affect their differentiation into multiple cell lineages including EC that reshape the surrounding pathological GC stroma, hence MSC probably directly give rise to EC to contribute to GC vascular network (65–67). Additionally, experimental evidence shows that MSC are able to give rise to CAF with the activation of TGF-β derived from GC-exosomes (68). CAF are well-demonstrated stromal cells and are often coordinated and overlapping with GC-MSC in reprogramming tumor itself and the stroma vicinity to contribute to cancer invasion and migration, acquired chemoresistance and EMT (69–71). Hp infection in GC cells would therefore enhance the expression of hepatoma-derived growth factor (HDGF) which also accelerates the transition of MSC into CAF and amplify their synergistic effects (72). Former studies stressed the role of CAF in ECM remodeling that control the aggressiveness and metastasis of cancer cells (73, 74). However, newly data indicate that secretion of MMP-2, MMP-7, MMP-9 and MMP-14, matrix metalloproteinases needed for ECM degradation are also increased in MSC to destruct external barriers to facilitate GC invasion and migration (75).

## The Prospects of MSC in Targeted Therapy

### MSC Act as Drug Delivery Vehicles

With their inherent advantages, like tumor and metastases tropism, easy isolation, low immunogenicity, MSC are ideal vehicles for tumor-directed therapy to raise efficacy (**Table 1**). Recently, a novel polymer AHP-OA-FA has been employed to infuse with MSC that enhance the tropism into GC with increased drug concentration and aggregation in local tumor lesions than pure MSC, and the polymer exerts no impact on the surface marker, proliferative capacity and motility of MSC, thus could be serving as more potent carriers for targeted therapy (84). Prerequisites for application of MSC are that the therapeutic agents they transported can be released after reaching the tumor site and exhibit strong toxicity only to tumor tissues. Cai and coworkers (76) established immuneapoptotin-armored MSC which continually secrete immuneapoptotin e23sFv-Fdt-tBid and exhibit significantly killing effect to GC cells expressing HER2 *in vitro* study. Importantly, after being intravenously administered in HER2 reconstituted syngeneic mouse models, these primed MSC exhibit persistent localization at tumor areas, display markedly stronger immunoapoptotin staining and better anti-tumor effect in comparison with direct delivery of the purified immunoapoptotin or delivery by Jurkat cells, indicating that MSC mobility can be well extended for the specific killing of HER-2 overexpressed GC. Besides, MSC expressing a transgene encoding NK4, the antagonist of HGF receptors, were observed to migrate and accumulate in tumor tissue, and effectively inhibit GC growth *via* suppressing tumor angiogenesis as well as triggering tumor cell apoptosis (77). In much the same way, umbilical cord blood (UCB)-MSC being infected with lentivirus vectors carrying LIGHT (TNF receptor superfamily) genes are also reported to induce severer apoptosis of GC cells (78). These trails illustrate that MSC as vectors possess a large scope of complexity, including apoptosis-inducing genes, oncolytic viruses, cytotoxic agents and anti-angiogenic agents. To our interest, a recent study demonstrated that MSC could also be modified to carry hemoglobin genes and supply oxygen to GC cells to reverse the hypoxic microenvironment and reduce the resistance to cisplatin and 5-FU (79). However, there are still lacking in evidence to ensure that the engineered MSC do not cause tumor progression or recurrence after long periods of infiltrating in cancer stroma. The first prospective, uncontrolled, single-arm phase I/II study on MSC-based therapy using autologous genetically modified MSCs in advanced gastrointestinal adenocarcinoma (TREAT-ME1) has been finished and suggested that MSC_apceth_101 cells (a total dose of 3.0×10^6^ cells/kg) expressing herpes simplex virus tyrosine kinase (HSV-TK) combined with ganciclovir was safe and could be well tolerated (85, 86). While it is a small study with 10 patients and no GC cases included, thus future multicenter investigations with larger samples are warranted to realize the safe and effective transformation of MSC-based therapy into GC settings.

**Table 1:** Summary of applications of MSC in targeted therapy of GC.

Mechanism of action	Source of MSC	Anti-tumor compound	Effects	References
MSC as anti-tumor drug vectors	BM-MSC	Immunoapoptotin e23sFv-Fdt-tBid	Tumor growth↓	(76)
	BM-MSC	NK4: antagonist of hepatocyte growth factor receptors (Met)	Tumor necrosis↑; microvessel formation↓	(77)
	UCB-MSC	LIGHT(TNFSF14):TNF receptor	Tumor apoptosis↑	(78)
	BM-MSC	Hemoglobin genes (HBA2 and HBB)	Chemotherapeutic effect↑	(79)
Target the MSC recruitment	BM-MSC	AMD3100: inhibitor of CXCL12/CXCR4 signaling axis	Tumor growth↓; gastric dysplasia↓	(80)
	BM-MSC	SB225002: CXCR2 inhibitor	Tumor necrosis↑; growth↓; lymph node metastasis↓	(26)
Target the MSC-GC interactions	GC-MSC	Resveratrol	EMT↓; metastasis↓	(81)
	BM-MSC	Anti-IL-6 antibody Anti-IL-8 antibody Anti-CCL-5 antibody 17β- estradiol	Tumor invasiveness↓	(82–84)
	BM-MSC	Etomoxir (ETX): inhibitor of FAO	Cancer stemness↓; chemo-resistance↓	(35)
	GC-MSC	YAP shRNA	Tumor migration↓; invasion↓; pro-angiogenic ability↓	(41)
	GC-MSC	PDGF-DD siRNA or su16f	Tumor proliferation↓;migration↓	(42)
	GC-MSC	Curcumin	Tumor angiogenesis↓	(64)

### Target the Recruitment Course of MSC and the Downstream Vicious Signals

In most circumstances, the active recruitment of MSC commonly occurs ahead of GC initiation, especially in Hp-related carcinogenesis. Hp induced inflammation milieu is abundant in functional molecules such as TNF-α, TGF-β, CXCL12 and interferon γ that conducive to MSC recruitment for their tissue healing functions (28, 87–89). Corresponding to this point, Ruan et al. (27) labeled MSC with amino-modified FMNP that keep stable fluorescent signal and magnetic properties with 14 days to display out the early gastric cancer (EGC) area, not only being with the potential of imaging EGC, these MSC also could inhibit tumor growth markedly under alternating magnetic field irradiation. MSC aggregating at inflamed stomach further prompts gastric carcinogenesis mainly through EMT once they fail to repair (55, 90). Hence earlier Hp eradication or intercepting MSC recruitment would make a difference to suspend carcinogenesis and progression (**Table 1**). Prior study has manifested that the AMD3100, an inhibitor of the CXCL12/CXCR4 signaling axis, could block the transformation of MSC into α-SMA+ myofibroblasts and the recruitment of MSC, inhibiting tumor growth and the development of gastric dysplasia (80). Similarly, the administration of antagonists of the CXCL1/CXCR2 axis was found to block the MSC recruitment in the GC mice model, decreasing the size of tumors as well as the number of lymph node metastases (26). On the other hand, directly disrupt inter-communication between MSC and GC also offer novel insights for GC treatment (**Table 1**). Such as resveratrol, which could suppress and revert the pro-metastatic effect of GC-MSC *via* counteracting GC-MSC-mediated Wnt/β-catenin signaling of GC cells (81). Curcumin is a bioactive compound and found to abrogate the NF-κB signaling and VEGF production to attenuate the GC-MSC-triggered tumor angiogenesis (64). In addition to these, several researchers have successfully adopted specific neutralizing antibodies of IL-8, IL-6, and CCL-5 to inhibit MSC-mediated GC invasive motility, and 17β- estradiol also impair the functions of IL-8, IL-6, and CCL-5 under the same context *via* ceasing the activation of their downstream Src/Cas/Paxillin signaling pathway, thus hormonal therapy might be anticipated based on MSC activity (82, 83, 91).

## Conclusions

MSC, with their multi-lineage differentiation and immune privilege nature, have shared great popularity in regenerative medicine and allogeneic transplantation. As the stromal progenitor cells, their role in tumor progression and TME is being put under the spotlight of tumor researches. Most studies have confirmed the tumor-contributing role of MSC, while the anti-tumor effect of MSC gradually unveils in several cancer types such as melanoma; glioma; HCC (92–94). Also, there are opposite results clarifying that MSC from human adipose tissue and the umbilical cord could inhibit GC progression and induce apoptosis of cancer cells (95, 96). One convincing explanation of the discrepancy attaches importance on the process of reprogramming of tumor cells that convert naïve MSC, which often exert a divergent effect on tumor progression, into pro-tumorigenic educated tumor-associated MSC. However, other factors, like the differences among tumor models, the heterogeneity of MSC, the timing and dose of the MSC injected are also accepted to influence the process of MSC-cancer interactions and lead to inconsistent results [reviewed in (97, 98)].

This review stresses the reciprocal crosstalk of malignant cells and MSC in the progression of GC, which can partly account for the complexity and heterogeneity of tumor-stroma connections. GC and the secret mediators in the niche would induce MSC recruitment and educate them into cancer-associated MSC with stronger tumor-promoting potential. In response, through cell physical contact or secretomes, MSC could aberrantly motivate oncogenic signals, deregulate metabolic plasticity and elicit EMT in GC cells to promote proliferation, invasion, migration and chemoresistance. Indirectly, reprogrammed MSC can deliver their signals horizontally to non-tumor cells in the TME to boost their pro-tumor functions with the repression of local immune response, stimulation of tumor angiogenesis and ECM remodeling. Given that MSC homing occurs early in the precancerous stage, they can be used for the detection of EGC. And they are also ideal carriers to deliver anti-cancer agents to tumor lesions with their low immunogenicity and well-accommodation, an increased concentration and lethality of drugs in target tissues would be expected. While further researches are warranted to identify whether the tumor-promoting role of MSC would override the inhibiting effect from drugs they delivered. In addition, the reciprocal reprogramming of MSC and GC as well as their domino effect spread to the TME prove beneficial for tumor growth and progression, strategies intercepting these vicious signaling connections represent a hopeful prospect in GC treatment. Taken together, the reciprocal reprogramming of GC and MSC triggers more active tumor-supporting signals that sustain tumor progression and remodel the surrounding pathological stroma. The tumor tropism nature of MSC and their extensive roles in GC deserve more in-depth investigation as they earn promising targets for cutting-edge cancer treatments.

## Author Contributions

YL wrote the review. XZ and YZ were responsible for figure and legend and final editing. XL contributed to conceiving the concept, analyzing the article and preparing for submission. All authors contributed to the article and approved the submitted version.

## Funding

This study was supported by research grants from Zhejiang Provincial National Science Foundation of China (Grant no.LY16H160031).

## Conflict of Interest

The authors declare that the research was conducted in the absence of any commercial or financial relationships that could be construed as a potential conflict of interest.
